# Mechanisms of Aging in Bone Marrow Stem Cells

**DOI:** 10.1371/journal.pbio.0050215

**Published:** 2007-07-24

**Authors:** Liza Gross

Settling into middle-age typically brings the unpleasant realization that many things don’t work quite like they used to. Spicy foods cause gastric distress. Muscles lose tone and agility. Wounds take longer to heal. Theories of aging abound and genetics clearly plays some role, but there’s little disagreement that the body’s maintenance and repair systems deteriorate with age. Stem cells combat the aging process by replenishing old or damaged cells—particularly in the skin, gut, and blood—with a fresh supply to maintain and repair tissue. And if stem cells themselves are protected from aging, as has been proposed, then maybe scientists could find a way to bolster their ability to rejuvenate aging tissue.

Unfortunately, new evidence suggests that this regenerative capacity also declines with age as stem cells acquire functional defects. In a new study, Stuart Chambers, Margaret Goodell, and their colleagues investigated the molecular mechanisms underlying these deficits by using microarrays to analyze the gene expression profiles of aging hematopoietic stem cells (HSCs), the precursors of blood cells. The researchers found that genes involved in the inflammatory and stress response became more active with age, while genes important for regulating gene expression and genomic integrity became less active. These results lend strong support to the notion that HSCs succumb to the wear and tear of aging, just like other cells, and shed light on the mechanisms of aging.

Previous studies have shown that the number of progenitor cells from whole bone marrow increases with age compared with the number of adult marrow cells. To determine whether the same holds true for HSCs, the researchers needed to isolate pure populations of stem cells from bone marrow for analysis. To do this, they took advantage of the fact that so-called side population (SP) HSCs routinely discharge a standard laboratory dye that other cells retain. (Other HSC-isolating methods depend on cell-surface markers, but the researchers consider the SP strategy more reliable.) By examining SP cells taken from mice ranging in age from 2 to 21 months, the researchers determined that SP cells—which they confirmed were HSCs based on the presence of HCS-specific cell-surface markers—showed a dramatic increase in abundance with age.

**Figure:**
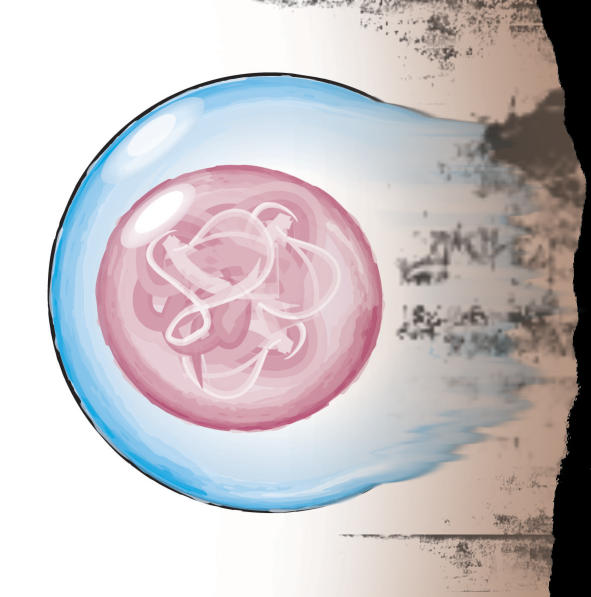
Aging HSCs exhibit a functional decline (yet an increase in cell number) and display a heightened stress and inflammatory response along with signs of epigenetic erosion. (Image: S. M. Chambers)

To study HSCs’ regenerative capacity over time, Chambers et al. isolated HSCs from young (aged 2 months) and old (aged 21 months) mice and then transplanted either young or old cells into mice whose bone marrow cells had been destroyed by radiation. The young and old HSCs gave rise to new marrow cells at roughly the same pace 4 weeks after transplantation. But at 8 and 16 weeks after transplantation, the old HSCs’ contributions had dropped considerably, suggesting that aging HSCs lose their repopulating capacity. Yet because HSCs increased in number, overall blood production from HSCs remained stable.

For insight into the molecular mechanisms underlying HSC loss of function over time, the researchers analyzed the expression profile of over 14,000 genes in HSCs taken from mice that were 2, 6, 12, and 21 months old. Transcription increased with age for 1,600 genes and decreased for 1,500 genes. Many of the “up-with-age” genes encode factors involved in either the inflammatory response or the stress response pathway that eliminates misfolded proteins. The “down-with-age” genes are mostly involved in processes that preserve genomic integrity, such as DNA repair and chromatin remodeling (chromatin is the protein matrix surrounding DNA).

The finding that genes involved in the inflammatory response are expressed more (called up-regulation) as HSCs age is consistent with evidence linking inflammation and aging in the kidney, brain, and arteries. It may also help explain why HSCs lose function. One of the up-regulated genes, *P-selectin*, encodes a cell surface adhesion molecule. Because transplanted HSCs depend on cell adhesion to colonize bone marrow properly, the researchers explain, inappropriate up-regulation of genes encoding P-selectin may interfere with this process.

The markedly reduced expression (or down-regulation) of genes involved in chromatin remodeling—an “epigenetic” regulator of gene expression—suggested that transcriptional activity might be dysregulated across the genome. This possibility was supported by the observation that expression patterns of genes located in the same chromosomal regions changed in a coordinated fashion over time. These coordinated transcription changes involved twice as many up-regulated genes as down-regulated genes, suggesting a global loss of transcriptional silencing. The finding that genes required for transcriptional silencing are down-regulated with age also supports this interpretation.

Though the dominant model attributes the physical effects of aging to an accretion of isolated genetic insults, these results link age-related decline to global mechanisms, operating across the genome. In the researchers’ “epigenetic view of aging,” chromatin dysregulation provides a logical explanation for the numerous and diverse age-related changes observed at the molecular, cellular, and organismal levels. Over the normal course of aging, chromatin dysregulation leads to dysregulation of many genes, which in turn leads to a loss of normal cellular functions and a loss of growth regulation. These changes ultimately increase the risk of cancer—which, in many of its forms, increases dramatically with age. Future studies can investigate how epigenetic regulation, inflammation, and the stress response interact to better understand the molecular mechanisms of aging, and why so many of us face a high risk of cancer in our later years.

